# Differential contributions of implicit and explicit learning mechanisms to various contextual cues in dual adaptation

**DOI:** 10.1371/journal.pone.0253948

**Published:** 2021-07-08

**Authors:** Maria N. Ayala, Denise Y. P. Henriques

**Affiliations:** 1 Department of Psychology, York University, Toronto, Canada; 2 Centre for Vision Research, York University, Toronto, Canada; 3 School of Kinesiology and Health Science, York University, Toronto, Canada; University of Münster, GERMANY

## Abstract

The ability to switch between different visuomotor maps accurately and efficiently is an invaluable feature to a flexible and adaptive human motor system. This can be examined in dual adaptation paradigms where the motor system is challenged to perform under randomly switching, opposing perturbations. Typically, dual adaptation doesn’t proceed unless each mapping is trained in association with a predictive cue. To investigate this, we first explored whether dual adaptation occurs under a variety of contextual cues including active follow-through movements, passive follow-through movements, active lead-in movements, and static visual cues. In the second experiment, we provided one group with a compensatory strategy about the perturbations (30° CW and 30° CCW rotations) and their relationships to each context (static visual cues). We found that active, but not passive, movement cues elicited dual adaptation. Expectedly, we didn’t find evidence for dual adaptation using static visual cues, but those in the Instruction group compensated by implementing aiming strategies. Then, across all experimental conditions, we explored the extent by which dual learning is supported by both implicit and explicit mechanisms, regardless of whether they elicited dual adaptation across all the various cues. To this end, following perturbed training, participants from all experiments were asked to either use or ignore the strategy as they reached without visual feedback. This Process Dissociation Procedure teased apart the implicit and explicit contributions to dual adaptation. Critically, we didn’t find evidence for implicit learning for those given instructions, suggesting that when explicit aiming strategies are implemented in dual adaptation, implicit mechanisms are likely not involved. Thus, by implementing conscious strategies, dual adaptation can be easily facilitated even in cases where learning would not occur otherwise.

## Introduction

While it has always been imperative for humans to learn to manipulate new tools, with the rise of modern technologies we are often required to operate and switch under multiple different visuomotor mappings. This ability to switch between different tasks accurately and efficiently is an invaluable feature to a flexible and adaptive human motor system. We can skillfully use a tool, correct for any movement errors while we use it, and then anticipate the consequences of switching to a completely different tool or environment. Often, we make large errors when first learning to move under novel circumstances, but our motor systems quickly adapt, such that eventually, and rather quickly, we produce smooth, accurate movements despite behavioral or environmental constraints.

To study the ability of the motor system to switch between learned visuomotor maps, variants of the same environment can be introduced serially (i.e., ABA paradigm) or concurrently (i.e., dual adaptation paradigm). ABA paradigms probe how an internal model can be affected by the subsequent learning of another, while dual adaptation paradigms have the advantage of challenging the motor system to learn, acquire, and switch between multiple internal models, mimicking its daily demands. Typically, when faced with unpredictable opposing perturbations to movement that vary from trial to trial, the motor system fails to adapt, or substantially improve, across many blocks of training, leading to interference [1–5]. However, adaptation to two or more visuomotor perturbations (i.e., dual adaptation) can proceed when each visuomotor map has been sufficiently cued during training, although what makes a cue sufficient is still unclear. Usually, the relevance of the cue to the task and possibly the “intrinsic” or motor-based nature of the cue appears to facilitate dual adaptation (e.g. [6, 7]), while cues that are more “extrinsic” or less pertinent to the task lead to mixed findings (e.g. [8–11]). Indeed, more recent work has now shown the critical role of motor planning over execution with regards to reducing interference between concurrently-learned visuomotor maps [12–15]. In one pivotal study by Sheahan and colleagues, using distinct follow-through movements as contextual cues, the researchers dissociated the contributions of planning and execution by manipulating the timing of the presentation of the follow-through targets [12]. To isolate the planning component, participants saw both initial (perturbed) and follow-through targets as they initiated their reach, but the follow-through target was extinguished to signal to participants that a follow-through motion is not required. Their main finding, that compensation occurs even in the absence of the execution of the follow-through, suggests that the key component in representing motor adaptation lies in action planning, and not in the act of executing a physically different limb state [12].

Adaptation to a single perturbation can be characterized by at least two qualitatively different learning mechanisms: [1] an explicit, or conscious process which arises due to the implementation of aiming strategies [16, 17], and [2] an implicit process which is thought to function without awareness, reflect adaptation of internal models in the cerebellum, and largely produce reach aftereffects [16, 18]. Explicit and implicit contributions can also be elicited when adapting to opposing visuomotor rotations for some contextual cues, such as when targets are displayed in different workplaces (a cue that usually leads to dual adaptation rates similar to that for single-rotation adaptation [8, 19]), and when the perturbations are large and blocked, as demonstrated by Schween and colleagues [15]. In one of their dual adaptation experiments, Schween and colleagues showed that overlapping strategies led to interference, but when provided with compensatory strategies, participants showed overall learning and reach aftereffects [15]. Still, it’s unclear to what extent these underlying processes are engaged, so here we break down the explicit and implicit contributions to dual adaptation across a diverse set of contextual cues.

In this series of experiments, we explore the efficacy of various cues and implementing explicit aiming strategies in dual adaptation. First, we looked at whether distinct, active follow-through movements and more complex, lead-in movements can facilitate adaptation to opposing visuomotor rotations. This has been similarly demonstrated under force-field paradigms [13, 20], but it’s unclear whether the active component of the contextual cue drove dual adaptation, so in the first experiment we isolated the active movement component (i.e. the self-generated follow-through motion) and replaced it with a passive visual-only follow-through that mimicked that motion. We found that both active movement cues elicited dual adaptation and were able to provide a stronger contextual cue compared to the passive movement cue.

Using a Process Dissociation Procedure (PDP; adapted from [21], we teased apart the implicit and explicit contributions to dual adaptation and found that implicit mechanisms were involved for cues that elicited dual learning. In the second experiment, we equipped a separate Instruction group with a compensatory strategy about the perturbations (30° CW and 30° CCW rotations) and their relationships to each context (static visual cues) and compared their performance to a Non-instructed group that experienced the same visual cues and their associated perturbations. We didn’t find evidence for dual adaptation given only static visual cues, but those in the Instruction group were able to compensate by implementing aiming strategies. Critically, we didn’t find evidence for implicit contributions for those who were instructed, but found that instruction played a role, suggesting that aiming strategies only elicited explicit mechanisms. Thus, by implementing conscious strategies, dual adaptation can be easily facilitated even when learning would not occur otherwise.

## Method

### Participants

One-hundred and thirty-five participants (94 females, 20.40 ± 3.23 years old, mean ± SD) were recruited and assigned to participate in the following experiments and were granted a bonus credit for either an undergraduate psychology or kinesiology course. All participants were right-handed, had normal or corrected-to-normal vision, and were naïve to the purpose of the experiment. All participants provided written, informed consent. Procedures were approved by the York University Human Participant Review Committee (HRPDC # e2017-267) and were in accordance with the declaration of Helsinki. Two participants withdrew from the experiment due to discomfort and were thus excluded from all analyses. Table 1 displays the demographic summary of participants.

**Table 1:** Demographic summary of experimental groups including sample size, mean and standard deviation of age, and sex (number of female participants).

Experiment	Group	N	Mean age (SD)	Sex (F)
1a	Active Follow-through	28	20.15 (3.64)	22
	Passive Follow-through	30	19.97 (2.22)	17
	CCW-only	10	23.50 (9.42)	8
	CW-only	10	20.60 (2.41)	9
1b	Active Lead-in	31	22.26 (7.09)	17
2	Non-instructed	12	19.53 (2.53)*	11
	Instructed	12	19.09 (0.94)	8

*One participant in this group did not disclose their age.

### Apparatus

**Fig 1:**
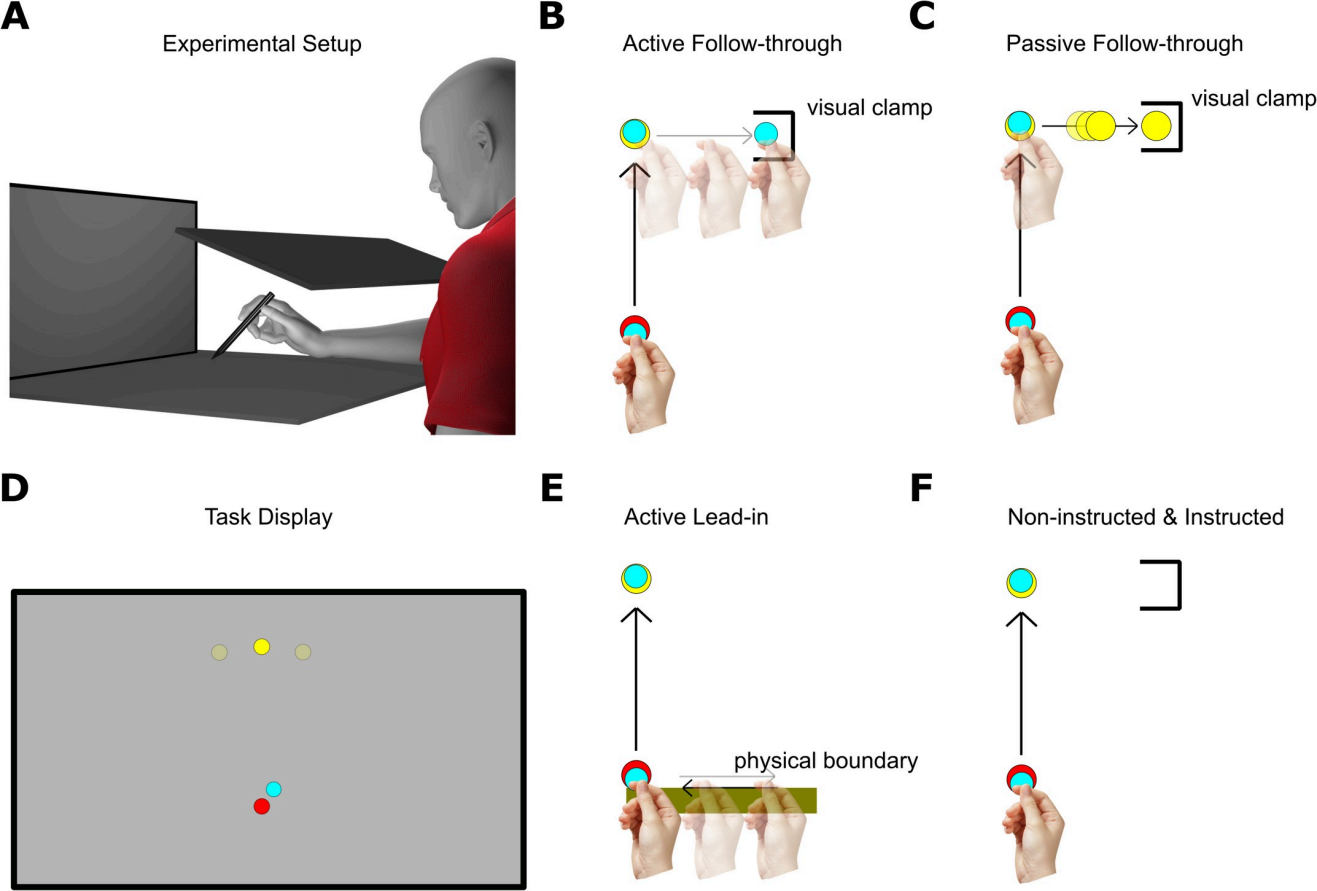
Experiment setup and task schematic. A. Participants reached along a horizontally-placed digitizing tablet. An opaque shield occluded the view of their hand. Participants viewed the motion of their hand-cursor on a monitor located at the end of the tablet. B. For Experiment 1a, those in the Active Follow-through condition reached to the yellow target disc, immediately followed by a visually-clamped reach to the box target. CW-only and CCW-only control groups completed the same task but only experienced a single perturbation. C. In the Passive Follow-through condition, participants reached to the yellow target disc, stopped, and only viewed the yellow target disc move to the target box. D. Participants saw targets discs located at one of three positions. E. For Experiment 1b, participants made an unseen reach along a solid edge to the left or right (brown bar), returned to the home position, and then to the yellow target disc, completing a three-part sequence. F. For experiment 2, those in the Non-instructed condition only reached to the yellow target disc, while they saw target boxes that indicated the perturbation in that trial. Those in the Instructed condition performed this same task but were instructed with a compensatory strategy.

Participants sat on an adjustable chair facing a digitizing tablet (Wacom Intuos3, 12” x 12” surface, resolution resampled by 1440 x 900 pixels at 60 Hz) and a vertically-mounted monitor (Dell Technologies, 22” P2217 LED screen) located approximately 55 cm from the horizontal tablet workspace. The tablet was placed at waist level so that hand movements were made along the horizontal plane (Fig 1A). To prevent participants from seeing their arm movements, an adjustable opaque shield was placed above the tablet [8, 22, 23]. All tasks required participants to reach to a target disc (1.5 cm in diameter) by moving a digitizing pen that moved a hand-cursor (1 cm in diameter) in a 1:1 ratio. Some tasks required a secondary reach from the target disc to a final target box (3 x 3 cm, e.g. Fig 1B). The target disc was radially-spaced and located at either 75°, 90°, or 105°, at a distance of 10.5 cm from home position. In all experiments but one, the target disc was flanked by a target box located 7.5 cm to its left or right, but never both at once.

### General procedure

In Experiment 1a (“Active and Passive Cues Experiment”), we examined how active and passive contextual cues vary in their efficacy in facilitating dual adaptation to opposing visuomotor rotations. In the first group (“Active Follow-through”), we associated distinct follow-through movements with opposing visuomotor rotations (Fig 1B). In the second group (“Passive Follow-through”), we removed that follow-through motion and instead associated a distinct visual target consequence to each visuomotor rotation (Fig 1C). That is, instead of asking participants to move to the target boxes (follow-through), they only reached to the target discs, then simply viewed that target disc directly move into the target box. In Experiment 1b, we implemented lead-in movements to cue the perturbations: participants made an unseen leftward or rightward reach, then back to the home position, and finally to the target disc, completing a three-part motion (Fig 1E). Two groups, “CW-only” and “CCW-onl**y**” served as control groups and learned only a *single* perturbation (thus, under only *one* context; Fig 1B).

To better understand the explicit and implicit contributions in dual adaptation, we ran a second experiment (“Instruction Experiment”) where in one condition (“Instructed”) participants were not only told about the nature of the perturbation and the relationship of the perturbations to the target boxes, they were also given a compensatory strategy. For this instructed condition, participants merely viewed the same target boxes located to the left or right of the target discs (as in the Active Follow-through and Passive Follow-through groups); no movements to the target boxes were required (Fig 1F). In the other condition (“Non-instructed”), left and right target boxes were coupled with different perturbations, but no instructions were provided and no secondary movement to the target boxes occurred; in other words, this condition experienced a purely static visual cue (Fig 1F). Across all conditions, the presence of a left-flanking box was predictive of a 30° CCW rotation while the right-flanking box was predictive of a 30° CW rotation.

All participants started at the same home position and were told to make smooth and direct reaches to the target disc. Although there were no hard time restraints, participants were encouraged by the experimenter to move in a quick and uniform pace across training and were warned on screen if they moved toward the target too slowly. For trials involving visual feedback of the hand-cursor (also referred to as “closed-loop trials”), participants simply reached and attained the target disc. For trials involving no visual cursor feedback (also referred to as “open-loop trials”), participants reached towards the target disc with their unseen cursor and remained stationary for 500 ms until the home position appeared. To facilitate returning to the home position and proceed to the next trial during open-loop trials, a line on the home position appeared following target acquisition and changed in orientation depending on the position of the pen. A solid edge was also placed just below the home position to help participants find the home position. Likewise, visual feedback of the hand-cursor appeared when participants were within a 2 cm radius of the home position.

**Fig 2:**
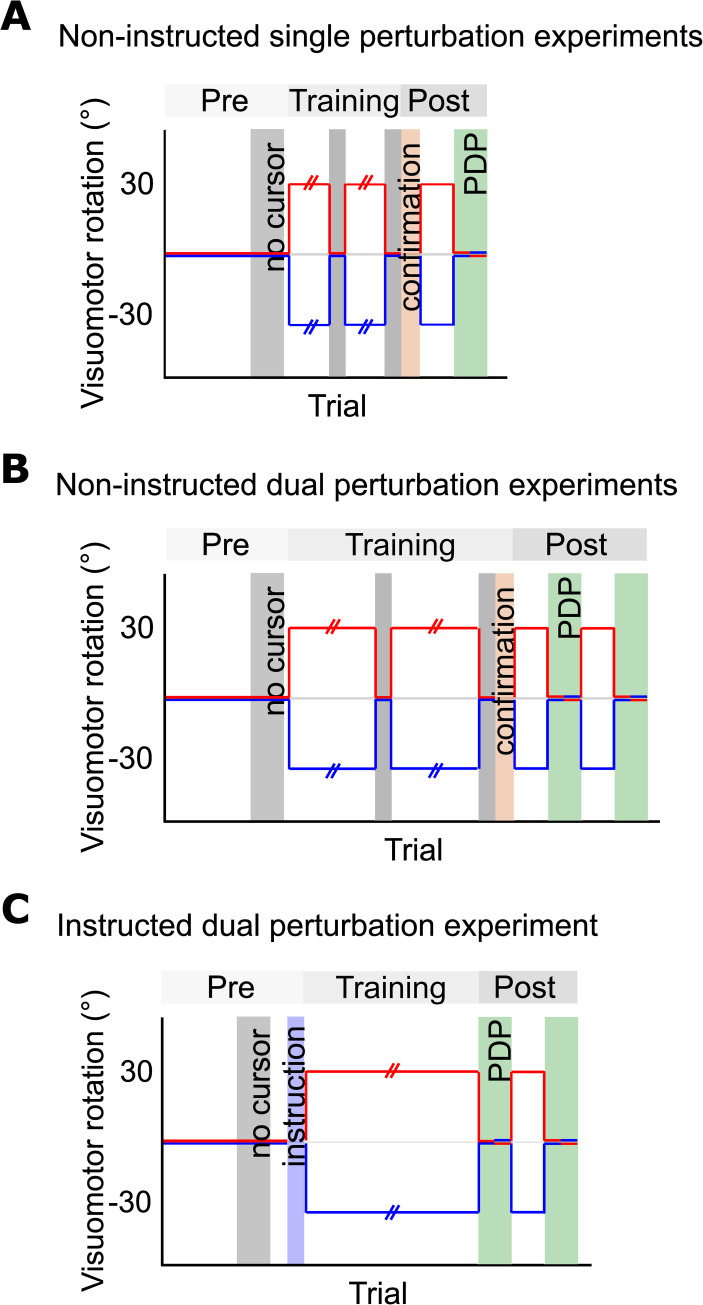
Perturbation schedule. Experiments consisted of Pre-training, Training, and Post-training periods. Pre-training captured baseline reaching movements, void of any perturbations. Grey bars depict blocks where participants do not receive visual feedback of the cursor (No-cursor). During training, perturbations were introduced depending on the condition. Post-training involved PDP tests (green bars) interleaved with additional training (perturbed) blocks to preserve any learning. A. Non-instructed single perturbation experiments. Two groups follow this perturbation schedule: CW-only and CCW-only control groups. B. Non-instructed dual perturbation experiments. Four groups fall into this perturbation schedule category: Active Follow-through, Passive Follow-through, and Active Lead-in. The Non-instructed condition from Experiment 2 also completed this protocol. During Training, participants experienced interleaved trials with CW and CCW rotated cursors. B. Instructed Group (Experiment 2) session schedule. Only the Instructed group in the Instruction Experiment followed this perturbation schedule. All PDP trials, shaded in green, were counterbalanced.

Across all groups, participants completed pre-training, training, and post-training sessions (Fig 2). For the CW-only and CCW-only control conditions, participants completed the task in approximately one hour (Fig 2A), while the rest completed the task in approximately two hours (Fig 2B and 2C). Apart from the Instructed condition in Experiment 2, participants were not informed about the presence or nature of the rotation at the start of the experiments. After the experiment, participants answered a series of questions to assess their awareness of the rotation and the experimental objectives.

### Experiment 1a: Active and passive cues experiment

Experiment 1a consisted of 4 groups: [1] Active Follow-through [2], Passive Follow-through [3], CCW-only control, and [4] CW-only control.

### Active follow-through group

Twenty-eight participants completed the Active Follow-through condition (Fig 2B). Participants reached towards the target disc followed by a reach to either its LEFT or RIGHT to a target box (Fig 1B). After acquiring the target disc (Fig 1B, yellow disc), the hand-cursor was visually clamped to only show the horizontal component of movement (i.e., only show movement along the x-axis) as they moved towards the target box (Fig 1B, left-opened black box). Considering findings by Howard and colleagues [20], we minimized the dwell time at target disc acquisition and encouraged participants to try to make their reach and follow-through movements as continuous as possible.

#### Pre-training (baseline measures)

The aim of the pre-training session was to obtain baseline performance and familiarize participants with the task. Participants reached towards the target disc 60 times with visual feedback of the aligned cursor (veridical), followed by 24 reaches with no visual feedback of the cursor (i.e., No-Cursor) to record baseline open-loop reach errors (Fig 2B, Pre-training).

#### Rotated training (adaptation)

The aim of reach training was to expose participants to a visuomotor rotation in order to assess the learning rate and later, reach aftereffects. Participants reached 360 times with interleaved 30° CW and CCW rotated cursors followed by 12 “Within-Training” No-Cursor reaches (Fig 2B, Training). This was repeated for a total of 720 rotated training trials. During training, each rotation was associated with either a LEFT or RIGHT target box (Fig 1B) to determine whether dual adaptation can occur when cued by different movement contexts. At the beginning of every trial, participants saw both the initial (Fig 1B, yellow disc) and final targets (Fig 1B, left-opened black box). CW and CCW trials were presented in a pseudo-randomized order such that participants encountered all three possible targets for each cursor rotation before any target location was repeated.

#### Post-training (analysis for awareness)

Immediately after Training, we confirmed with participants that the cursor would not move in the same direction as their hand, and that they needed to compensate and keep that compensatory strategy in mind (Fig 2B, Confirmation, in orange) as they will later be prompted by the screen to either use a “strategy” (Include-Strategy) or *not* use a “strategy” (Exclude-Strategy). This Process Dissociation Procedure (PDP; adapted from [21]) allowed us to make inferences about the participants’ awareness, which in turn allowed us to tease apart implicit and explicit contributions to dual adaptation. After Confirmation, participants reached with both CW- and CCW-rotated cursors for 24 trials to preserve any dual adaptation. This was followed by 12 Include-Strategy No-Cursor trials, then 12 Exclude-Strategy No-Cursor trials (Fig 2B, Post-training). Finally, they again reached with both CW- and CCW-rotated cursors for 24 trials, followed by another 12 Include-Strategy trials and 12 Exclude-Strategy trials. All post-training tasks were counterbalanced.

### CW-only and CCW-only control groups

Twenty participants served as CW- or CCW-only controls for all dual groups. The task is identical to the Active Follow-through group, except participants in these groups experienced either a 30° CW *or* 30° CCW rotation of the cursor during training. The experiment began with a Pre-training session, consisting of 60 aligned reaches followed by 12 No-Cursor reaches.

#### Rotated training (adaptation)

During training, these participants reached 180 times with a rotated cursor (30° CW *or* 30° CCW) followed by 15 No-Cursor reaches twice (Fig 2A, Training). This was repeated for a total of 360 rotated training trials. Since participants in these groups only experienced a single perturbation, they only saw either a left or a right target box across training.

#### Post-training (awareness analysis)

As in the Active Follow-through group, immediately after Training we confirmed with participants that the cursor would not move in the same direction as their hand and that they needed to compensate for this (Fig 2A, Confirmation). After Confirmation, participants reached with CW- *or* CCW-rotated cursors for 24 trials to preserve any adaptation. This was followed by 12 Include-Strategy No-Cursor trials, then 12 Exclude-Strategy No-Cursor trials (Fig 2A, Post-training). All post-training tasks were counterbalanced.

### Passive follow-through group

Thirty participants completed the Passive Follow-through condition. Unlike the Active Follow-through group, participants simply had to reach to the initial target (disc), stay stationary on the disc, which initiates the movement of the disc into the final target (box; Fig 1C). Participants were told to stay on the initial target location until the disc has fully migrated into the final target box. When participants failed to remain still, a high-pitched beep and the visual message “Stay still!” signaled them to stop moving until all stimuli disappear.

Participants in the Passive Follow-through Group followed the same perturbation schedule as the Active Follow-through Group (Fig 2B).

### Experiment 1b: Active lead-in cue

In a follow-up experiment, we implemented a lead-in movement to cue the perturbations (Fig 1E). Thirty-one participants completed the Active Lead-In experiment. Instead of making a secondary movement to the box target, participants made a lead-in movement: first, an unseen initial reach to the left or right along a physical edge, whose direction is signaled by a black arrow superimposed on the home position, followed by a visually-clamped reach to the home position, then finally to the target disc (Fig 1E). Three participants fell in the extreme outlier range (i.e., errors beyond the interquartile range criterion) and were excluded from analysis.

The Active Lead-in condition followed the same perturbation schedule as the Active Follow-through and Passive Follow-through conditions (Fig 2B).

### Experiment 2: Instruction experiment

Experiment 2 consisted of 2 conditions: the [1] Instructed, and the [2] Non-instructed group.

### Instructed group

Twelve participants completed the Instructed condition. While these participants saw the static visual cues (target boxes), they did not reach towards the boxes or see any visual motions toward the boxes; they simply had to reach to the target disc and stay stationary (Fig 1F). When they failed to remain still, a high-pitched beep and the visual message “Stay still!” signaled them to stop moving until all stimuli disappear. Critically, these participants were instructed about the nature of the perturbations and their relationship to the static visual cues (target boxes). They were explicitly given a strategy on how to counter the perturbations.

Fig 2C shows the perturbation schedule for those in the Instructed condition. During Pre-training, participants reached towards the initial and final targets 60 times with visual feedback of the aligned cursor, followed by 24 reaches with no visual feedback of the cursor (Fig 2C, Pre-training). After Pre-training, participants were informed about the nature of the rotations and were provided with a strategy to counteract the rotation so that they can move the cursor in a straight path to the targets (Fig 2C, Instruction, shaded in blue). More specifically, they were instructed to visualize the location of the home position as the center of a clock face. They were told that for trials where they saw the box on the left side, reaching towards a number on the clock would result in the cursor heading an hour behind, so they should aim an hour ahead to make a straight path to the target. They were told that the opposite was true for trials where they saw the box on the right side. Full instruction scripts can be found in this experiment’s OSF repository (https://osf.io/v2hwp/).

During training, participants reached 720 times with interleaved CW- and CCW-rotated cursors (Fig 2C, Training). Post-training followed the same protocol as in the Active Follow-through, Passive Follow-through, and Active Lead-in conditions (Fig 2C, Post-training).

### Non-instructed group

Twelve participants completed the Non-instructed condition. This experimental group served as controls for the Instructed group; participants only saw the final target and were not informed about the rotations, the coupling between the rotation and target box, nor were they given any compensatory strategy (Fig 1F). Participants reached to the target disc and stayed stationary (and were otherwise alerted as described above in the Instructed condition if they failed to do so). Fig 2B shows the perturbation schedule for the Non-instructed condition. Pre-training, training, and post-training followed the same protocol as in the Active Follow-through, Passive Follow-through, and Active Lead-in conditions (Fig 2B).

### Analysis

Cursor movement data was digitally smoothed using a first-order, low-pass Butterworth filter with a frequency cut-off of 2.5 Hz. Movement onset was fixed at 10% of peak velocity. The experimental data was analyzed offline using R (preprocessing and analysis scripts can be found in https://github.com/ayalamar/sequence), using kinematic error (operationalized as angular deviation at maximum velocity) during training and No-Cursor trials. Our main error measure, angular error at maximum velocity, refers to the angular difference of the target and cursor at peak velocity relative to the home position. Other error proxies for trials with visual feedback of the cursor include cursor path length, which refers to the length of the cursor movement in centimeters, and maximum angular deviation, which refers to the maximum angular difference of the target and the cursor (relative to the home position) along the cursor trajectory. For trials without visual feedback of the cursor, along with angular deviation at maximum velocity, we also looked at endpoint angle which refers to the angular difference of the target and the cursor (relative to home position) at the end of the reach (target-disc acquisition). For all our analyses involving blocks, we averaged across blocks of 3 trials to include a movement to each target. The assumed level of significance for all statistical tests was α = .05, and post-hoc comparisons were Bonferroni-corrected.

Angular error was subject to tests of normality, and corrections for unequal variances were applied where necessary. When the normality assumption was not met, we instead applied non-parametric tests (i.e., Wilcoxon rank-sum and signed rank tests for t-tests). Finally, post-hoc power analyses were completed for key comparisons. All raw and processed experimental data can be found on the project’s OSF (https://osf.io/v2hwp/).

#### Visuomotor adaptation

Classic analysis of visuomotor adaptation in dual adaptation studies typically includes comparisons between single-perturbation groups and dual-perturbation groups to compare rates of learning. However, as it has been shown in the literature, dual adaptation rarely parallels the learning rate to that of single adaptation, so we opted to report only dual learning statistics for brevity. For those who are interested, omnibus tests are available on the project OSF.

The overall difference in reaching error between the first trial and the last block reflect the extent of learning. Since CW and CCW rotations were not expected to elicit different rates of learning, we analyzed normalized errors, but we provide visualizations of signed errors to show whether compensation occurs in the expected directions. A planned repeated-measures t-test (first block vs. final block; one-sided) was used to show whether adaptation has occurred within each experimental group. To further visualize learning and provide a more intuitive measure of effect size, we compared performance from the beginning of rotation training (*Initial block error*) to the final block of experiencing the rotation (*Final block error*), for each rotation. We quantified this Percent Improvement (PI) for experiments 1a, 1b, and the Non-instructed condition for experiment 2:

$$Percent\;Improvement=\frac{Initial\;block\;error-Final\;block\;error}{Initial\;block\;error}\times 100$$

Positive PIs indicate reduction in errors over time while negative PIs indicate worsening of errors over time. PIs for the Instructed condition were calculated relative to the size of the rotation, since some participants show near-perfect compensation at the initial block of training that yield inflated PIs. In a follow-up analysis, we collapsed across active conditions from Experiment 1 and compared their PI to that of the Passive Follow-through condition using a Wilcoxon rank-sum test.

#### Reach aftereffects

An alternative measure to test whether adaptation has occurred is the presence of reach aftereffects during No-Cursor trials. Reach aftereffects refer to rotation-dependent, deviated reaching in the absence of visual feedback of the cursor. Using the PDP [21], reach deviations can be analyzed to segment explicit and implicit components. It is thought that implicit learning is reflected in No-Cursor trials where participants are told not to implement any strategies in their movements (i.e., Exclude-Strategy No-Cursor trials). To this end, we subtracted any biases from the final block of No-Cursor trials following Pre-training from the first Exclude-Strategy No-Cursor trial and determined whether this was significant from zero using a one-sample t-test (one-sided) for all experimental groups.

Include-Strategy No-Cursor trials consist of not only explicit, but also implicit processes. Thus, to determine whether participants were using a compensatory strategy, we compared the reach deviations for the first Include-Strategy No-Cursor trials and the first Exclude-Strategy No-Cursor trials, after accounting for baseline biases, using a repeated-measures t-test (one-sided) for all experimental groups.

#### The role of instruction

In this final set of analyses, in Experiment 2 we set off to understand the role of explicit strategy in dual learning by comparing the results between the Instructed group and the Non-instructed group. To confirm that participants in the Instructed condition could implement the strategy, we compared their first block of training to those from the Non-instructed condition using a Welch independent-measures t-test.

First, to assess whether any implicit learning occurs following training, we ran a 2 X 2 Mixed Repeated-Measures ANOVA comparing reach aftereffects across Pre-training No-Cursor tasks and Exclude-Strategy No-Cursor tasks between the Instructed and Non-instructed groups. A significant effect of No-Cursor task would indicate the presence of reach aftereffects even when participants are excluding strategy, suggesting the presence of an implicit learning mechanism.

Then, to examine the effect of instruction, we ran a 2 X 2 Mixed Repeated-Measures ANOVA comparing across Exclude-Strategy No-Cursor tasks and Include-Strategy No-Cursor tasks between the Instructed and Non-Instructed groups. If participants are aware and are using the instructed strategy, we would see a significant difference between Include-Strategy No-Cursor trials (in the expected directions) and Exclude-Strategy No-Cursor trials. Finally, this analysis will be followed by planned comparisons within each group (Instructed and Non-instructed groups) to determine whether having instructions significantly affects the magnitude of reach aftereffects.

## Results

To investigate the underlying mechanisms behind dual adaptation, we implemented various contextual cues in the first experiment, and provided detailed instructions on how to counter the perturbations given the contexts in the second experiment. In the first experiment, those in the Active Follow-through and Active Lead-in conditions made active movements that cued the perturbations either after or before the perturbed reach, respectively. Those in the Passive Follow-through condition reached to the target disc and only saw that target move to a secondary target whose location cued the perturbation. In the second experiment, one group was given a compensatory aiming strategy to dual adapt (Instructed group), while another group was not given any strategies (Non-instructed group). Across all experiments, a leftward sequence (or visual location of the box) was indicative of a 30° CCW rotation, while a rightward sequence (or visual location of the box) was indicative of a 30° CW rotation.

**Fig 3:**
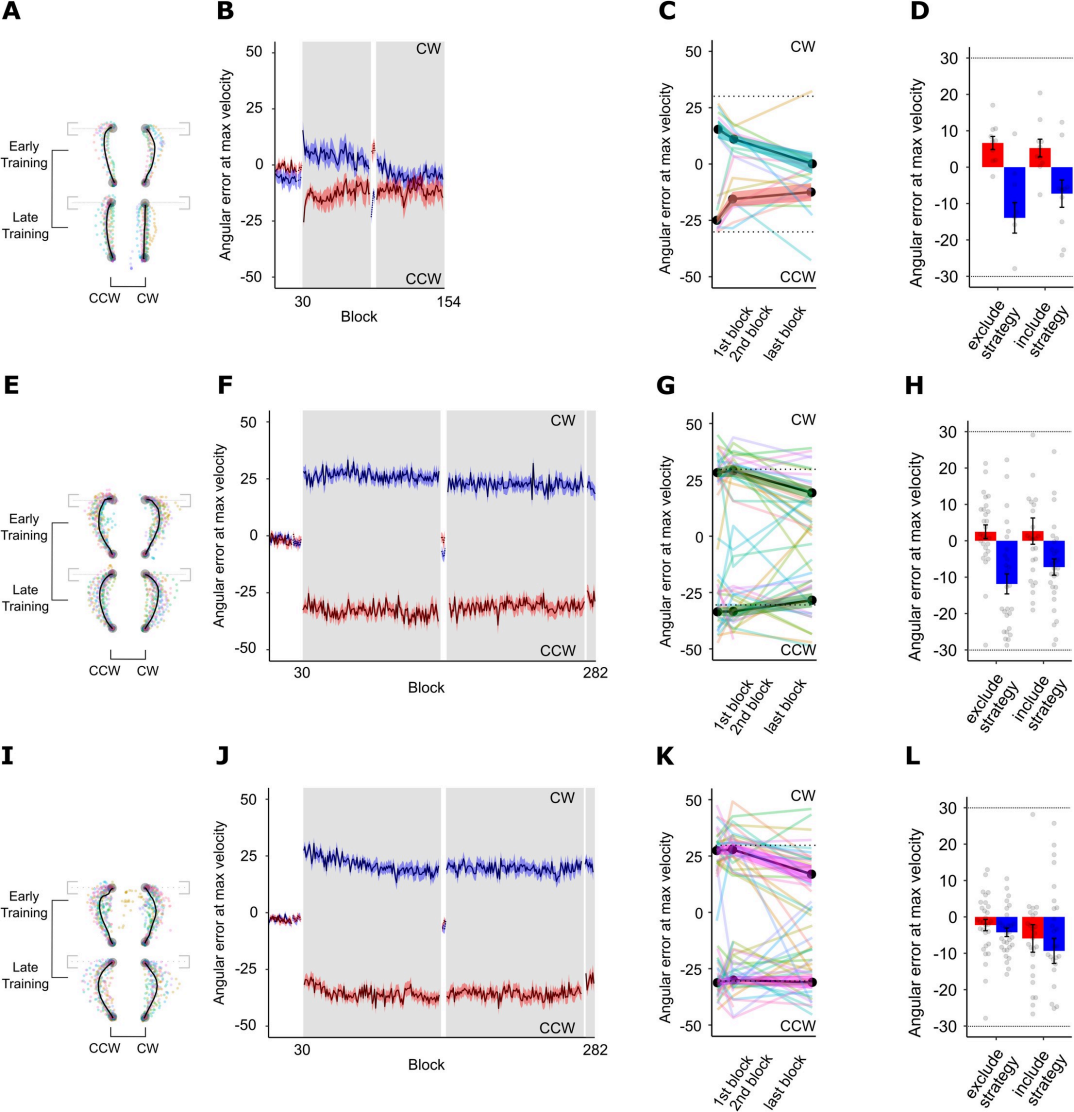
Experiment 1a performance. A. Averaged hand-cursor trajectories for CW- and CCW-only control groups. Coloured translucent dots represent individual trajectories. B. Visuomotor adaptation across all training blocks for CW- and CCW-only control groups. The learning curve for the CW-only group is in blue, and the CCW-only group in red. C. Truncated visuomotor adaptation across training for CW- and CCW-only control groups. Colored translucent lines represent individual learning curves for CW- and CCW-only control groups. D. Reach aftereffects in post-training for CW- and CCW-only control groups. Grey dots represent individual participant errors. Red bars represent CW errors and blue bars represent CCW errors. Dashed lines represent rotation magnitude experienced during training (30° CW and 30° CCW). Error bars represent SEM. E. Averaged hand-cursor trajectories for the Active follow-through group. F. Visuomotor adaptation across training blocks for the Active follow-through group. G. Truncated visuomotor adaptation across training for the Active follow-through group. H. Reach aftereffects in post-training for the Active follow-through group. I. Averaged hand-cursor trajectories for the Passive follow-through group. J. Visuomotor adaptation across training blocks for the Passive follow-through group. K. Truncated visuomotor adaptation across training for the Passive follow-through group. L. Reach aftereffects in post-training for the Passive follow-through group.

Participants of Experiment 1a in the CW-only and CCW-only control groups exhibited typical visuomotor adaptation since they only encountered one perturbation, with large errors approximately the size of the rotation at the beginning of training that gradually reduced to near baseline levels (Fig 3B) and rotation-dependent reach aftereffects (Fig 3C).

### Visuomotor adaptation

#### Experiment 1a: Active and passive cues experiment

**Fig 4:**
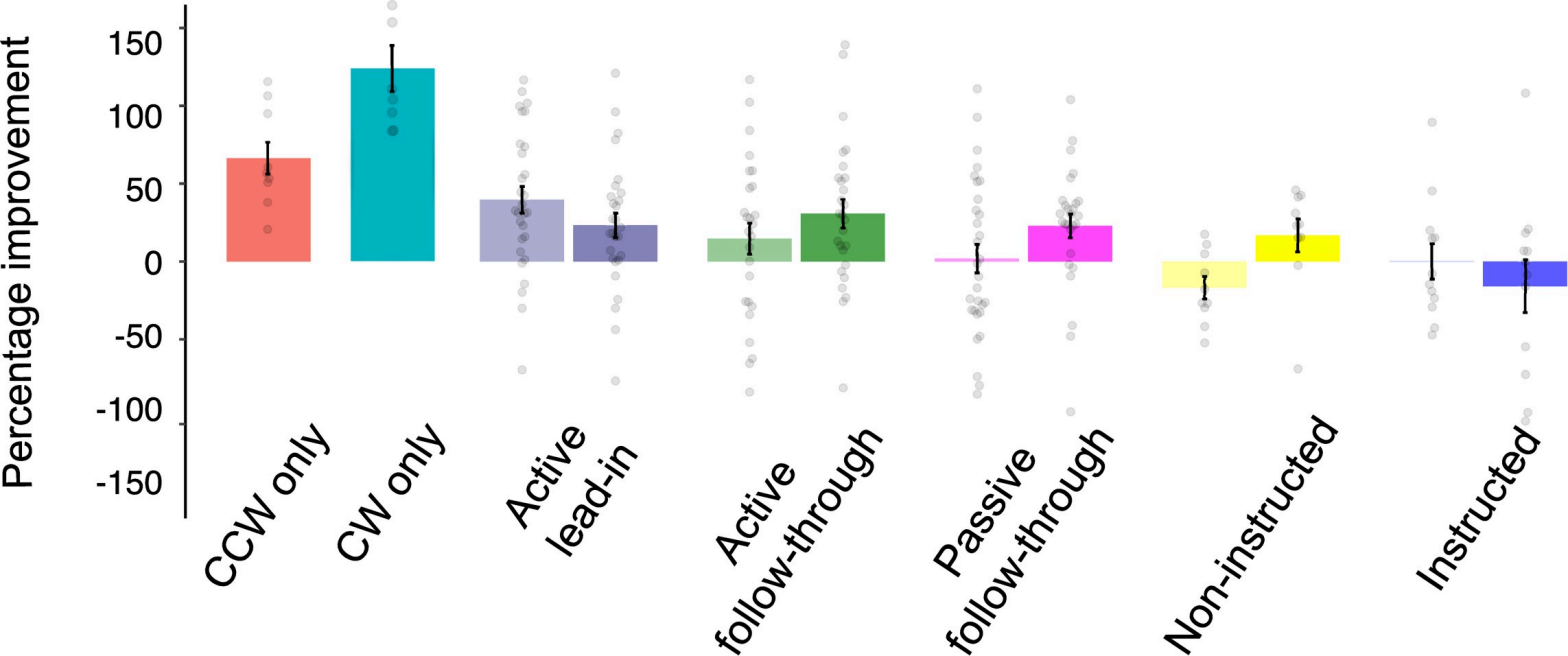
Percent improvement across training for all experimental groups and rotations. For dual conditions, bars with a darker hue represent PI for CW trials, and vice-versa. Grey dots represent individual improvement scores. Positive values reflect improvement, and vice-versa. Error bars represent SEM.

*Active follow-through group*. Participants made a two-part, active follow-through movement involving a reach to the target disc followed by a secondary, visually clamped reach to the target box. Fig 3F and 3G (green) shows the extended and truncated learning curves for Active Follow-through group, respectively. We saw a small, significant reduction in reaching errors at maximum velocity across training (t(27) = 2.07, p = 0.02, one-tailed, d = 0.39). Similarly, cursor path length reduced across training (t(27) = 4.24, p < 0.001), along with maximum deviation along the cursor trajectory (t(27) = 2.52, p < 0.01). To intuitively show the effect of the cue and individual performance, Fig 4 depicts percent improvement per experimental group and per rotation. Distinct active follow-through cues led to improved performance for both rotations as seen in Fig 4 (green bars); on average, participants improved across training by a modest 14.72% for the CCW rotation and 30.81% for the CW rotation. Post-hoc analysis of errors at maximum velocity revealed that for a moderately sized effect (d = 0.50) and our modest sample size, we had obtained power of 0.72 for this condition.

When we removed visual feedback of the cursor but kept the cues intact, we found significant reach aftereffects in their expected directions (t(27) = 4.06, p < 0.001, one-tailed, d = 0.77; Fig 3H, exclude strategy), also indicated in endpoint angles significant from zero (t(27) = 4.49, p < 0.001, d = 0.85). When asked to implement a compensatory strategy, we did not find a significant difference in reach errors relative to those during Exclude-Strategy No-Cursor trials (t(27) = -0.80, p = 0.78; Fig 3H, include strategy); endpoint angle was similarly not significant from 0 (t(27) = -0.88, p = 0.81). These findings further support that distinct active follow-through motions can facilitate dual adaptation, but we found that this compensation was largely implicit.

*Passive follow-through group*. In contrast to the Active Follow-through condition, in the Passive Follow-through condition participants were required to reach to the target disc with a CW- or CCW-rotated cursor, and then simply observe their target disc move towards the target box. Training performance is illustrated in Fig 3J and 3K (pink). This cue was insufficient at facilitating dual adaptation as suggested by large reaching errors that did not significantly reduce across training (t(29) = 0.91, p = 0.18), also reflected in their percent improvement which was close to zero (Fig 4, pink bars). Using a two one-sided equivalence test (TOST) with *a* = 0.05 and equivalence bounds of *d* = -0.50 and *d* = 0.50 signifying a moderate effect size, we found that the observed effect is statistically not different from 0 (t(29) = 0.911, p = 0.370) and statistically equivalent to 0 (t(29) = -1.828, p = 0.039). Similarly, maximum cursor deviation did not reduce over time (t(29) = 1.65, p = 0.05), although path length decreased over time (t(29) = 4.20, p < 0.001). Expectedly, reach aftereffects were not in the expected directions, although significant from baseline as participants erroneously compensated (t(29) = 1.86, p = 0.04); Fig 3L). Endpoint angle was not significant from baseline (t(29) = 0.87, p = 0.20). Furthermore, comparing Exclude-Strategy and Include-Strategy No-Cursor trials, we did not find a significant difference in reach error (nor were they in the correct directions) for both the error at maximum velocity (t(29) = -1.09, p = 0.86) or at the end of the movement (t(29) = 0.21, p = 0.42). Thus, merely viewing the movement sequence did not elicit dual adaptation.

#### Experiment 1b: Active lead-in group

**Fig 5:**
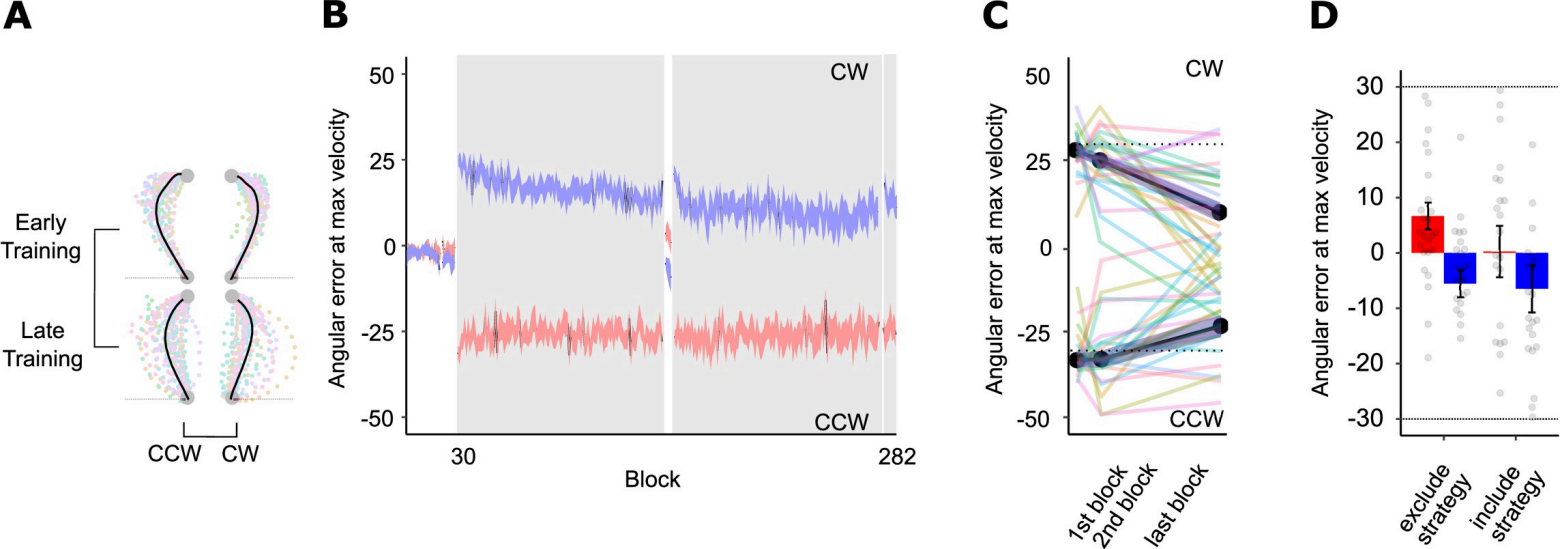
Experiment 1b performance. A. Averaged hand-cursor trajectories for the Active Lead-in group. Colored translucent dots represent individual trajectories. B. Visuomotor adaptation across training blocks. The learning curve for CW trials is in blue, and CCW trials in red. C. Truncated visuomotor adaptation across training for the Active Lead-in group. Colored translucent lines represent individual learning curves. D. Reach aftereffects in post-training. Grey dots represent individual participant errors. Red bars represent CW errors and blue bars represent CCW errors. Dashed lines represent rotation magnitude experienced during training (30° CW and 30° CCW). Dashed lines represent the rotation magnitude. Error bars represent SEM.

To explore whether a more complex lead-in movements can elicit dual learning, we had participants make a three-part movement sequence where the final portion of the reach was perturbed but the preceding motions were predictive of that perturbation. In the Active Lead-in group, participants reached along an edge from the home position, back to the home position, and finally, to the target disc. This active lead-in cue was able to elicit significant dual learning over training (t(26) = 3.44, p < 0.001, one-tailed, d = 0.66; Fig 5B, and 5C). Likewise, cursor path length reduced significantly over time (t(26) = 5.46, p < 0.001), but maximum deviation did not (t(26) = 0.41, p = 0.34), but this may have been driven by a few outlying participants. On average, participants reduced their errors by 26.95% for the CW rotation and 46.30% for the CCW rotation (Fig 4, purple bars). Post-hoc power analysis of errors at maximum velocity revealed that for a moderately sized effect (d = 0.50) and our modest sample size, we obtained a power of 0.28 for this condition.

Failing normality assumptions (W = 0.96, p = 0.02)), a follow-up Wilcoxon Rank-sum test indicated that active cues (Active Follow-through and Active Lead-in) tend to elicit larger PIs compared to passive cues (Passive Follow-through; W = 1049, p = 0.01, *δ* = 0.30).

Reach aftereffects for the Active Lead-in group were in the expected directions (Fig 5D). When asked to exclude any explicit strategy used, participants showed significant reach aftereffects in their expected directions, suggesting the involvement of an implicit mechanism (t(29) = 2.95, p = 0.003, d = 0.54). Endpoint angle was not significantly different from 0 (t(29) = 0.68, p = 0.25), but this was likely driven by an outlying participant. Conversely, when asked to include a strategy, participants did not show any additional reach deviations in the expected directions as would be expected if they had used a strategy (t(29) = -0.97, p = 0.83); endpoint angle mirrored these findings (t(29) = -1.27, p = 0.89).

#### The role of instruction in dual adaptation

**Fig 6:**
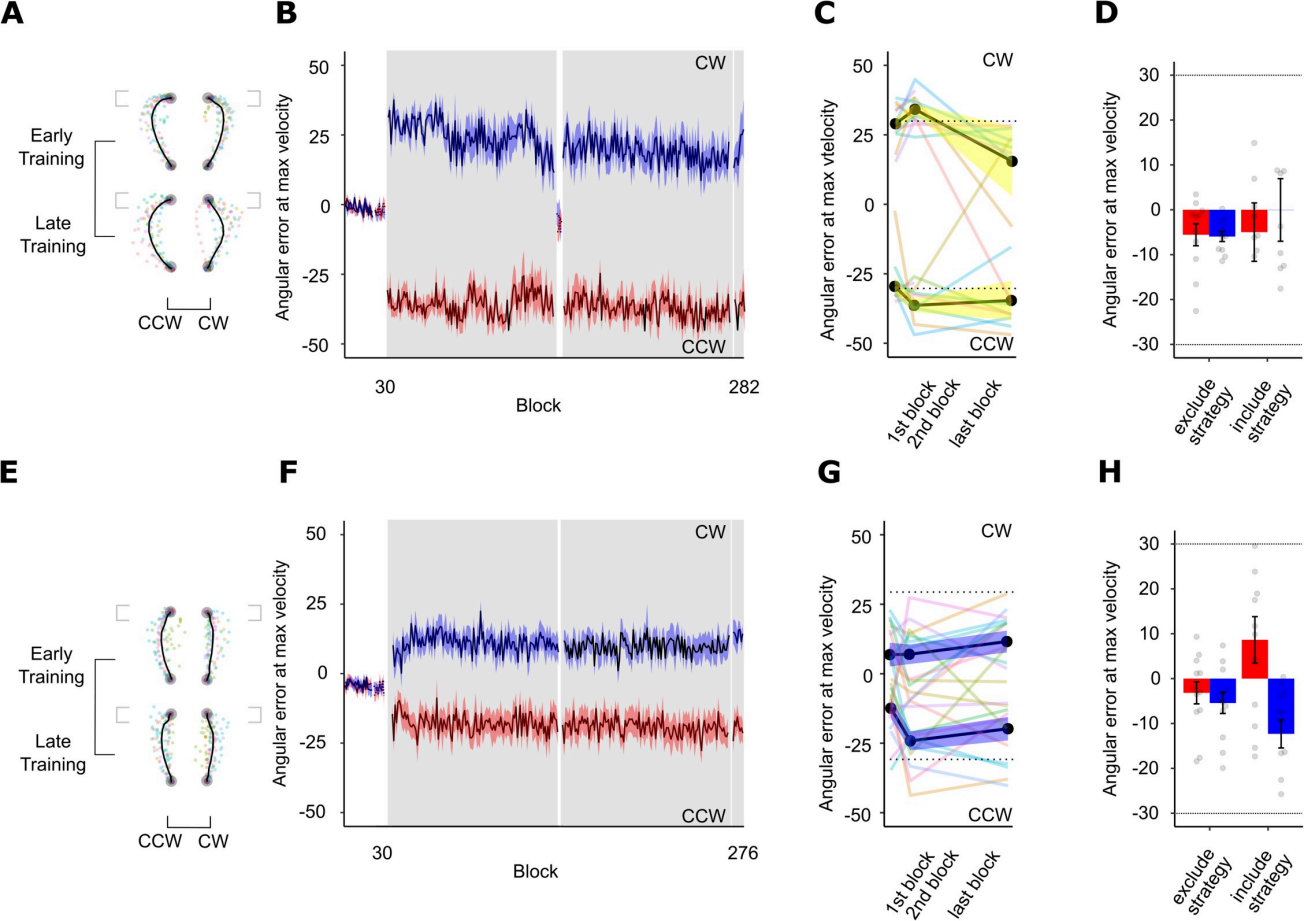
Experiment 2 performance. A. Averaged hand-cursor trajectories for the Non-instructed group. Coloured translucent dots represent individual trajectories. B. Visuomotor adaptation across training blocks for the Non-instructed group. The learning curve for CW trials is in blue, and CCW trials in red. C. Truncated visuomotor adaptation across training for the Non-instructed group. Colored translucent lines represent individual learning curves. D. Reach aftereffects in post-training for the Non-instructed group. Grey dots represent individual participant errors. Red bars represent CW errors and blue bars represent CCW errors. Dashed lines represent rotation magnitude experienced during training (30° CW and 30° CCW). Dashed lines represent the rotation magnitude. Error bars represent SEM. E. Averaged hand-cursor trajectories for the Instructed group. F. Visuomotor adaptation across training blocks for the Instructed group. G. Truncated visuomotor adaptation across training for the Instructed group. H. Reach aftereffects in post-training for the Instructed group.

To examine the explicit and implicit contributions to dual adaptation, we instructed a group about the nature of the perturbations and how to compensate for them given the contextual cue (Instructed condition) and compared their performance to a group who completed an identical task but were not provided any instruction (Non-instructed condition). In the Non-instructed condition, we eliminated any active or passive sequences; participants saw the box targets but only reached towards the target discs. Expectedly, reaching errors at maximum velocity did not reduce across training (V = 28, p = 0.81; Fig 6B, and 6C), with both cursor path length (t(11) = 0.05, p = 0.48) and maximum angular deviation mirroring this finding (t(11) = -2.15, p = 0.97). On average, for those who only received a static cue, percent improvement was -16.85% for the CCW rotation and 16.76% for the CW rotation which suggests that participants failed to adapt using this visual cue (Fig 4, yellow bars).

Participants in the Instructed condition showed immediate benefits of strategy-use such that they made smaller errors right at the beginning of training. On average, those given instructions only had a 14.98° error in the first block of training, which is significantly smaller than that of the Non-instructed group at 32.54° (t(18.02) = -7.72, p < 0.001, d = 3.04). Failing normality assumptions (W = 0.68, p < 0.001), a Wilcoxon Rank-sum test on maximum deviation mirrored these results (W = 34, p = 0.03), but path length did not differ (t(16.00) = 0.68, p = 0.51) again likely due to two outlying participants. This advantage was fleeting, however, as participants in the Instructed condition did not further reduce their reaching errors across training (t(11) = -1.78, p = 0.87), suggesting that instruction benefits were immediate and not moderated by more training (Fig 6F and 6G); this was reflected in the maximum deviation measure (V = 47, p = 0.28) but not in path length (t(11) = 2.79, p < 0.01). Those in the Instructed condition did not show any improvement over time; their average reaching error by the end of training was only 17.98°, compensating for only about 40.07% of the rotation, implying that the role of instruction was limited and exerted most of its effects in the early stages of learning.

Comparing No-Cursor trials across the Instructed condition (Fig 6H) and Non-instructed condition (Fig 6D), we found no implicit learning such that Exclude-Strategy No-Cursor trials were no different from Pre-Training No-Cursor trials (F(1,22) = 0.00001, p = 0.99); this was similarly reflected when comparing endpoint angles from Pre-Training and Exclude-Strategy trials across groups (t(39.68) = -1.85, p = 0.07). In particular, using a TOST (a = 0.05, lower bound *d* = -0.50 and upper bound *d* = 0.50), we saw that the observed effect in the Non-instructed condition was not statistically different from 0 (t(11) = -0.0703, p = 0.945) and statistically not equivalent to 0 (t(11) = 1.662, p = 0.0624). Likewise, the Instructed condition similarly show indeterminate outcomes; the observed effect was not statistically equivalent to 0 (t(11) = -0.0634, p = 0.475) and not statistically different from 0 (t(11) = 1.669, p = 0.123). Further studies are thus needed to sufficiently support that implicit processes are not involved during Instructed or Non-instructed dual adaptation.

To probe the effect of instruction, we compared Exclude-Strategy and Include-Strategy No-Cursor trials between Instructed and Non-instructed conditions and found that having instructions significantly elicits more deviated reaches in the expected directions (angle at peak velocity F(1,22) = 17.71, p < 0.001, *η*_*G*_^2^ = 0.19; endpoint angle F(1,22) = 11.86, p < 0.001, *η*_*G*_^2^ = 0.20). Together, these findings suggest that when contextual cues convey no information, the motor system may instead rely on an explicit mechanism to support dual adaptation.

To assess whether explicit processes are engaged within *each* experimental group, we compared Include-Strategy No-Cursor trials with Exclude-Strategy No-Cursor trials for the instructed and non-instructed groups separately. For the Instructed condition, we found that explicit mechanisms are engaged such that Include-Strategy and Exclude-Strategy No-Cursor reaches significantly differed (angle at peak velocity t(11) = 3.24, p < 0.01, one-sided, d = 0.94; endpoint angle t(11) = 2.32, p = 0.02, d = 0.67; Fig 6H). Expectedly, we did not find the same for the Non-instructed group (angle at peak velocity t(11) = -0.09, p = 0.54; endpoint angle t(11) = 0.39, p = 0.35; Fig 6H). Using a TOST with *a* = 0.05 and equivalence bounds of *d* = -0.50 and *d* = 0.50 signifying a moderate effect size, we saw that the observed effect was not statistically different from 0 (t(11) = 0.756, p = 0.465) and statistically not equivalent from 0 (t(11) = -0.976, p = 0.175), yielding undetermined outcomes. Altogether these findings suggest that when given an explicit compensatory strategy to dual adapt, the motor system likely relies on explicit processes.

## Discussion

To better understand what allows for dual learning, we investigated various contextual cues including both actively-generated and passively-viewed follow-through motions. In another experiment, we explicitly provided participants with a compensatory strategy. Across all these conditions, we teased apart the explicit and implicit mechanisms underlying dual adaptation by separating the influence of strategy. We found that active movement cues facilitated dual adaptation, while passive cues did not. These simple active follow-through and lead-in movements were largely learned without awareness since we found implicit but not explicit contributions in their reach deviations during PDP trials. Expectedly, we didn’t find evidence that static visual cues convey useful information to the CNS, instead finding interference. However, when given instructions about the rotations and their relationships to the static visual cues, dual learning was able to proceed but only in the form explicit learning, as we didn’t find evidence for the presence of reach aftereffects attributed to implicit processes.

### The role of active vs. passive cues in dual learning

Previous work on dual adaptation has largely demonstrated that motor-based or “intrinsic” cues tend to be more successful at reducing interference and facilitating learning [6, 8, 15, 24–27]. We further explored this idea by investigating whether self-generated movement sequence cues, which are motor-based by nature, can facilitate dual adaptation. In both our Active Follow-through and Active Lead-in groups we found significant dual adaptation as evidenced by reduced reaching errors and significant reach aftereffects in the former experiment. Our findings support the force-field work by Howard and colleagues, who found that distinct follow-through motions can sufficiently cue the motor system to compensate for opposing force-field perturbations [13]. Other force-field work showed that peripherally-moving visual cues do not facilitate learning, while passively-viewed lead-in visual cues do [7, 20]. Thus, it was unclear if follow-through movement cues needed to be self-generated to facilitate dual adaptation. To explore whether the *active* lead-in or *active* follow-through was the key component in this contextual cue, we removed that active component of the cue, as in the Passive Follow-through condition and found no reduction in reaching errors or any reach aftereffects in the expected directions. This suggests that even dynamic visual cues—likely extrinsic like that used in the Non-instructed condition, are insufficient even when it is temporally linked as a consequence of the perturbed reach.

Research by Howard and colleagues also found that lead-in motions can be coupled with opposing force-fields to produce significant reach aftereffects [20]. They found that in their most effective dwell condition cued by a distinctive lead-in motion, participants were able to compensate up to 75% of the necessary force to overcome the perturbation. With these findings in mind, we designed the Active Lead-in condition to have minimal dwell times at the home position (100 ms) and found that participants compensated for less than half the size of the rotation in the Active Lead-in condition. There are a few factors that may account for this. One key difference is the complexity of our lead-in cue—participants were asked to make a three-part motion in which the final reach was perturbed. So, not only did participants in the Active Lead-in condition have to access the past motion as a cue (second lead-in motion to the home position), they also had to go one step back to access the motion cue prior to that (first lead-in motion from the home position). Thus, one possibility is that cues that take longer to execute hampers its efficacy. Indeed, Howard and colleagues have found that a complex combination of lead-in and follow-through motion cues elicited dual adaptation to opposing force-fields, but only after intensive training across 5 days [13]. Additionally, in a previous study we saw that doubling the training (from 360 to 720 trials) did not sufficiently induce greater improvement nor larger reach aftereffects [6], making it possible that there is a maximum level of dual adaptation for a specific cue. Thus, future work should investigate if, and to what extent, longer sequences elicit dual learning. An alternative and less prevalent account might be that certain cues are more effective at facilitating dual adaptation to more dynamic tasks (i.e., force-field perturbations), and less to visuomotor rotations like we saw in our present experiments. Together, these may account for a proportion of why we saw a smaller mean level of compensation compared to previous force-field work implementing similar cues.

Interestingly, knowledge about the perturbations alone does not seem to dictate whether dual adaptation will occur. While not reflected in *more deviated* Include-Strategy No-Cursor reaches, post-experimental interviews revealed that a large proportion of those in the Active Lead-in condition were aware of the perturbations (post-experimental responses can be found in the project’s OSF). In fact, in the Active Lead-in group, nearly 42% (13 out of 31) of participants were able to explain the nature of the perturbations and their relationships with the cues, compared to 8 out of 28 participants (28.57%) in the Active Follow-through group. This didn’t seem to lead to any advantage as both *active* experiments achieved a similar level of percentage improvement. For the experiments where dual adaptation did not occur, only one participant in the Non-instructed group and an intriguing 8 out of 30 (26.67%) participants in the Passive Follow-through group were able to explain the nature of the perturbations and their relationships to the cues. These post-hoc observations highlight the critical role of how explicit and implicit components of motor learning are assayed [28]. In a recent set of experiments, Maresch and colleagues found that more frequent sampling of explicit components via verbal report amplified explicit contributions, and that verbal report yielded larger explicit components compared to that of exclusion tests (i.e., PDP, as we adapted here) when matched for reporting frequency. Here we saw that post-experiment, some participants accurately report the nature and contextual associations of the perturbations, but not necessarily make the compensations *during* training. Possibly, this may be due to explicit mechanisms consisting of subcomponents, some of which are accessible depending on whether re-aiming is simply cached or recalculated [29], with our PDP measures likely placing greater emphasis on the latter. We should also recognize that explicit knowledge of the perturbation altogether does not necessarily imply that participants were obliged to make corrections [30]. We could have possibly detected a performance effect where learning occurred in the background but was not manifest in behavior. Lastly, minute differences in how participants are informed about the perturbation have the potential to influence the differential contributions of these sub-mechanisms. Thus, future work should assess these key differences in determining the optimal way on how explicit learning can be extracted from behavior. It should be also noted that some differences lie between conditions and it’s unclear how these factors affect our findings; for instance, the Active Lead-in condition involved a motion that elicited a movement cue toward the starting position, while the Active Follow-through condition elicited a movement cue around the target disc. Thus, it seems that even with a sizeable portion of participants having knowledge about the nature of the perturbation, we still do not see significant strategy application. Possibly, insufficient cues might require the implementation of explicit aiming strategies to be successful.

An alternative explanation has been put forth by Sheahan and colleagues [12], who showed that distinctive motor plans, not movement execution, allow for concurrent adaptation to opposing force fields. They found that participants still compensated in the direction depending on the follow-through target even when they did not execute a follow-through motion, suggesting that the representation of the motor plan drives this dual adaptation (action selection), not the follow-through movement itself (action execution) [12]. This provides an alternative explanation to the lack of dual adaptation in our Passive Follow-through condition, where it was unlikely that participants produced a motor plan relevant to the cue, since they only viewed a visual consequence of their movement. Our finding that passive cues don’t lead to significant dual learning provide further support to their proposition that dual adaptation is more likely to be associated with a distinctive internal state which is separate from the neural dynamics involved during execution [12].

Use-dependent learning can alternatively explain how those in the Active Follow-through and Active Lead-in conditions were able to adequately adapt to both rotations. Here, use-dependent learning can alter the learning process since perturbations were applied in a redundant dimension of the movement [31]. Since leftward-flanking lead-in and follow-through motions were always paired with CCW perturbations, it’s possible that participants developed a bias toward repeated movement directions (i.e., by always aiming to the right-hand side).

### Insufficient cues can be salvaged by aiming strategies

As expected, static visual cues did not show any predictive strength, so in addition we provided participants in the Instructed condition with aiming strategies that depended on that context. This implementation led to significant learning which suggests that when cues are insufficient (e.g., static visual cues), the CNS can tap into explicit mechanisms to support dual learning. This purely explicit learning, however, comes at a cost. We saw that those in the Instructed condition could immediately compensate by implementing the aiming strategy, but they failed to improve, only retaining a small portion of the aiming strategy by the end of the training session, and later reflected in the lack of implicit reach aftereffects. In contrast, using a strongly predictive cue of target workspace separation, Schween et al. [15] nicely demonstrated significant implicit reach aftereffects in their group that received verbal instructions about how to counter their blocked 60° CW and CCW rotations [15] that were otherwise shown to interfere. Indeed, we found a similar effect whereby dual adaptation as cued by separated target sets were able to elicit reach aftereffects that decreased in size as a function of the distance between workspaces [8]. Critically, we show here that moderately predictive cues can indeed elicit implicit learning, but in the case of the Non-instructed and Instructed groups, an ineffective cue even in conjunction with aiming strategies will be unsuccessful.

Finally, it should be emphasized that our Instructed group are by proxy provided some explicit component of learning (i.e., through the aiming strategy), while those in Non-instructed groups (e.g. in [15]) were able to derive it from visuomotor learning alone. In fact, Schween et al. (2018) found that participants had relatively decent estimates of the rotations, estimating over half of the rotations’ magnitudes in their explicit judgment task, while our explicit reach aftereffects in the Instructed group only reached about a third of the size of our rotations. In light of these differences, these findings bring up the critical point that explicit strategies can support dual learning but only to a limited extent. Here we found that explicit strategies can aid an impoverished context, while more recent work by Schween and colleagues have found more interactive contexts can elicit dual learning [32]. There is also the potential that the mechanisms behind dual adaptation differ for smaller and larger rotations. Finally, it remains unclear how rotation presentation schedules influence this dual learning (i.e., pseudo-randomized trial-by-trial vs. alternating blocks of 8 trials per rotation).

### Limitations

Post-hoc power analyses revealed that limited statistical power due to our modest sample sizes may have played a role in limiting the significance of some of the statistical comparisons we conducted. While we were able to obtain a power of 0.71 for our Active Follow-through condition, still shy of the recommended level of 0.80, we would have needed a sample size of approximately 64 in our Active Lead-in condition. Relative to the literature, Experiment 2 had an adequate sample size but due to its exploratory nature, future experiments looking at the role of instruction in dual adaptation should approach it with a larger N, with sizes at least comparable to those in Experiment 1; this need was additionally confirmed by our findings from equivalence testing.

## Conclusion

Altogether our findings show that while intrinsic cues (such as active lead-in and follow-through cues) provide an avenue for the motor system to learn two or more visuomotor maps at the same time, it is single-handedly the underlying explicit mechanism that drives learning when cues are insufficient. This has significant implications in short-term rehabilitation where time is constrained and the motor system must rely on explicit processes to re-learn multiple motor skills, so future studies should investigate how to enhance this explicit process so that learning can be retained over extended periods of time.
